# Effect of Adjuvant Treatments on Recipient Vessel Diameter for Free Flap Breast Reconstruction Using Computed Tomographic Angiography Analysis

**DOI:** 10.3390/medicina62020265

**Published:** 2026-01-27

**Authors:** Jong Yun Choi, Ahran Kim, Junhyeok Lee, Daiwon Jun, Jiyoung Rhu, Pill Sun Paik, Jung Ho Lee

**Affiliations:** 1 Department of Plastic and Reconstructive Surgery, Seoul St. Mary’s Hospital, College of Medicine, The Catholic University of Korea, Seoul 06591, Republic of Korea; jongparry@naver.com; 2 Department of Plastic and Reconstructive Surgery, Bucheon St. Mary’s Hospital, College of Medicine, The Catholic University of Korea, Bucheon 14662, Republic of Korea; anani0206@gmail.com (A.K.); saylee1231@naver.com (J.L.); jundw430@gmail.com (D.J.); 3 Department of Surgery, Bucheon St. Mary’s Hospital, College of Medicine, The Catholic University of Korea, Bucheon 14662, Republic of Korea; jyses82@naver.com (J.R.); pillsun@gmail.com (P.S.P.)

**Keywords:** microsurgery, free flap, breast cancer, breast neoplasms, internal mammary artery, thoracodorsal artery, radiotherapy, chemotherapy, angiography

## Abstract

*Background and Objectives*: The quality of recipient vessels is critical for successful microsurgical breast reconstruction, and iatrogenic damage should be minimized. Adjuvant radiotherapy (RTx) and chemotherapy (CTx) are widely used for breast cancer and may induce structural changes in recipient vessels. This study aimed to evaluate changes in recipient vessel diameters for breast reconstruction after adjuvant treatment in patients with breast cancer. *Materials and Methods*: A total of 167 patients with unilateral breast cancer who underwent surgical resection between 2017 and 2021 were retrospectively reviewed. Patients were classified into four groups: mastectomy only without adjuvant treatment (group A, *n* = 33), adjuvant RTx only (group B, *n* = 44), adjuvant CTx only (group C, *n* = 43), and combined adjuvant CTx and RTx (group D, *n* = 47). Preoperative and postoperative computed tomography angiography was used to measure the diameters of the thoracodorsal artery (TDA) and internal mammary artery (IMA) on the affected and unaffected sides. Differences in vessel diameters between sides and among groups were analyzed. *Results*: In groups B and D, the diameters of the affected TDA and IMA were significantly decreased compared with the changes observed on the unaffected side (*p* < 0.001). In contrast, there were no significant differences in vessel diameters between the affected and unaffected sides in groups A and C (group A: *p* = 0.644; group C: *p* = 0.367). *Conclusions*: Recipient vessel diameters for microsurgical breast reconstruction significantly decreased in patients who received postoperative RTx, with or without CTx. Plastic surgeons planning delayed breast reconstruction should be aware of these adjuvant therapy-related changes in recipient vessels and consider preoperative imaging assessment to accurately counsel patients regarding surgical risks and to support informed decision-making.

## 1. Introduction

Advancements in microsurgery have increased the success rate of free flap reconstruction post-breast cancer surgery. However, surgical failures due to vascular issues and other intraoperative complications persist. The condition of the recipient site, including that of the recipient blood vessels, is crucial in free flap reconstruction. In breast reconstruction, the most typically used vessel recipients are the internal mammary artery (IMA) and thoracodorsal artery (TDA), which yield excellent results with no significant differences [1].

Therefore, factors such as vascular calcification, vessel diameter, and hemodynamic status should be considered when assessing the condition of blood vessels. This is because severe calcification signifies a very small diameter of the blood vessel and poor hemodynamic status, which can lead to a poor success rate of the free flap. Furthermore, the diameter and quality of blood vessels are critical during surgery and postoperative recovery [2,3,4,5,6,7]. In many cases, breast cancer treatments involve combined radiation therapy and chemotherapy pre- and postoperatively. Previous studies have shown that these treatments can cause vascular damage increased susceptibility to multiple complications during free flap reconstruction [8]. Nonetheless, the effects of chemotherapy and radiation therapy on changes in blood vessel diameter remain unclear.

Recent advances in microsurgical technique and preoperative vascular imaging have improved the safety of autologous breast reconstruction, yet intraoperative challenges related to compromised recipient vessels remain a major concern [9]. Breast cancer patients frequently receive multimodal adjuvant therapy, and contemporary systemic agents and radiotherapy protocols can influence both oncologic outcomes and vascular morbidity. Therefore, for optimizing surgical planning, a more detailed understanding of how adjuvant treatments affect the diameter and quality of recipient vessels.

In this study, we conducted a study to investigate the effects of chemotherapy and radiation therapy on the diameters of the IMA and TDA in patients receiving adjuvant treatment for breast cancer.

## 2. Materials and Methods

Our study participants were selected from 798 patients with breast cancer who underwent mastectomy between February 2017 and February 2021. The inclusion criteria were female sex and available chest CT angiography data pre- and post-mastectomy. The exclusion criteria were male sex, previous treatment for cancers other than breast, history of neoadjuvant chemo- or radiotherapy, bilateral breast cancer, prior breast plastic surgery, and lack of available chest CT angiography results before breast cancer treatment.

**Figure 1:**
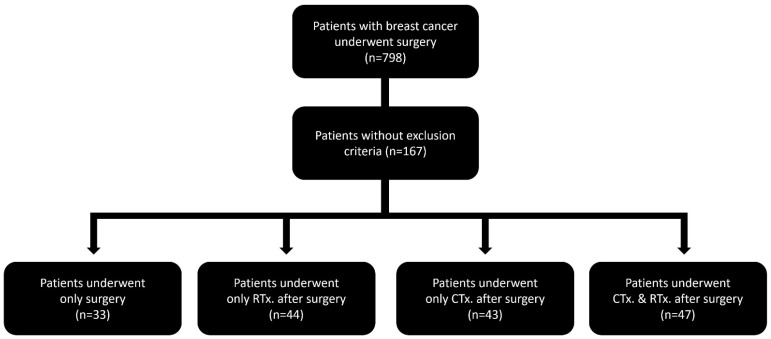
Patient classification Among the 798 patients with breast cancer who underwent mastectomy, 167 met the inclusion criteria. Of these patients, 33 underwent surgery alone, 44 received RTx postoperatively, 43 received CTx postoperatively, and 47 underwent CTx and RTx postoperatively. CTx, chemotherapy; RTx, radiation therapy.

Overall, 167 patients were included in the study. Among them, group A (*n* = 33) underwent breast cancer surgery alone without adjuvant radiation or chemotherapy, group B (*n* = 44) received only postoperative radiation therapy, group C (*n* = 43) received only postoperative chemotherapy, and group D (*n* = 47) received postoperative radiation and chemotherapy (Figure 1).

Radiotherapy was administered based on the hospital protocol, with a daily dose of 180 cGy for 28 days, resulting in a total dose of 5040 cGy. Chemotherapy was noted only as administered and did not include the specific method or procedure. In all patients who received chemotherapy, the postoperative CT angiography used for vessel measurements was performed after completion of chemotherapy, rather than during treatment, so the timing of CTA was consistently post-CTx.

**Figure 2:**
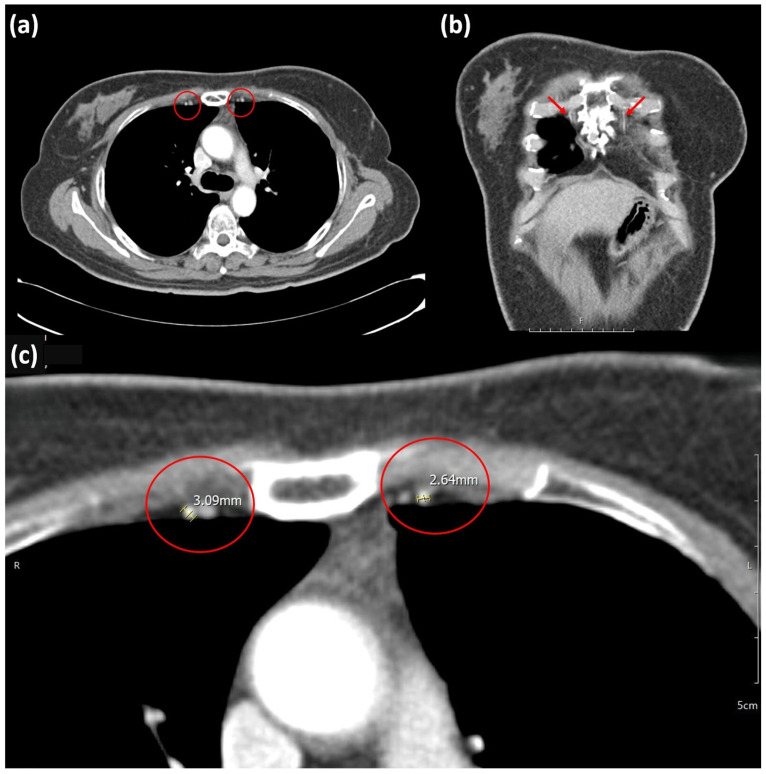
Measurement of the internal mammary artery in CT angiography. (**a**) Axial CT image with the red circles indicating the anatomical locations of IMAs. (**b**) Coronal CT image with red arrows highlighting the course of the IMAs. (**c**) Magnified axial CT image at a magnification of 491.91, with red circles indicating the measurement site of the right and left IMAs. CT, computed tomography; IMA, internal mammary artery.

**Figure 3:**
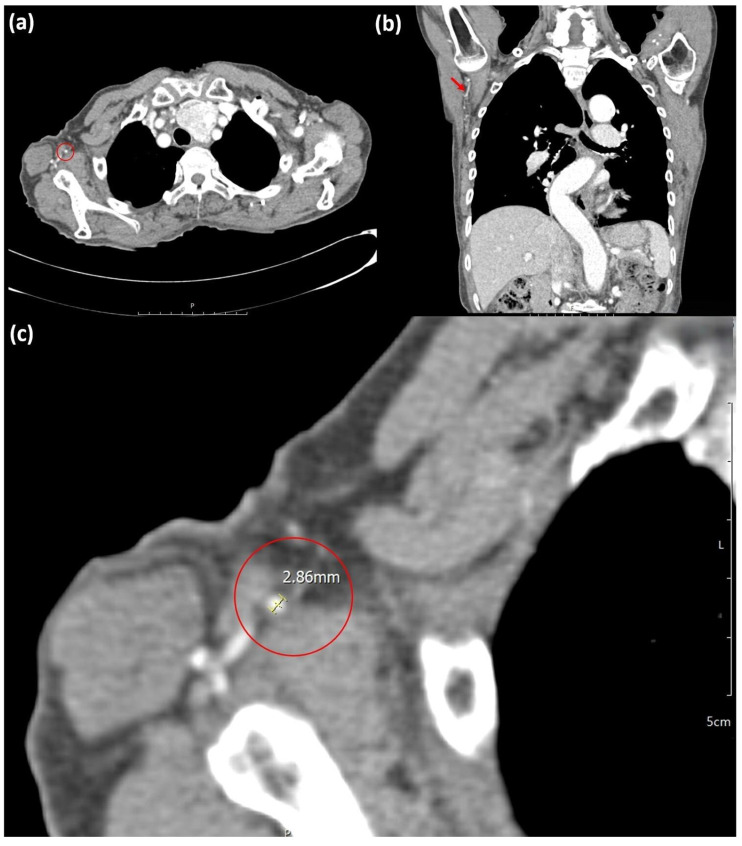
Measurement of the thoracodorsal artery on CT angiography. (**a**) Axial CT image with the red circle indicating the course of the TDA. (**b**) Coronal CT image with red arrows indicating the course of the TDA. (**c**) Magnified axial CT image at a magnification of 491.91, with the red circle indicating the measurement site of the TDA. CT, computed tomography; TDA, thoracodorsal artery.

Two independent plastic surgeons who are blinded to study details measured the diameters of the recipient vessels (TDA and IMA) on CT angiography images (Picture archiving and communication system (PACS); Maroview version 5.4, MAROTECH Inc., Seoul, Republic of Korea) on the affected and contralateral sides in the same patients. Based on previous research, the diameters of the IMAs were measured in the first and second intercostal space; each surgeon performed five measurements, and the mean measurement was recorded (Figure 2) [10]. The diameters of the TDAs were measured at their origin from the subscapular artery, as previously described (Figure 3) [10,11]. This view enlarged the CT cuts at the same ratio (×491.91). The reviewers then utilized the PACS program’s caliper function to measure each vessel diameter.

Institutional review board approval (IRB number: HC24RISI0023) was obtained, and a retrospective cohort study was conducted. This study was approved by the IRB of the Catholic Medical College. All data analysis was performed anonymously and followed the principles of the Declaration of Helsinki (1975, revised in 2013).

All continuous variables were expressed as mean ± standard deviation. Baseline characteristics in Table 1 were compared among the four groups using one-way ANOVA for continuous variables and chi-square tests for categorical variables. Within each group, pre- and post-treatment vessel diameters (ipsilateral and contralateral TDA and IMA) were compared using paired t-tests, which are appropriate for repeated measurements obtained from the same patients. For each group, the pre-to-post change in diameter (Δ diameter = post − pre) was then calculated separately for the ipsilateral and contralateral sides, and these two change values were compared using two-sample t-tests to evaluate whether adjuvant treatment produced asymmetric diameter changes between the operated and non-operated sides. For all tests, 95% confidence intervals were reported to quantify the magnitude and precision of the estimated effects, and a two-sided *p*-value < 0.001 was considered statistically significant. All analyses were performed using SPSS for Windows, version 24.0 (IBM Corp., Armonk, NY, USA).and the statistical plan was reviewed by the Department of Mathematics and Statistics, the Catholic university of Korea.

## 3. Results

The average age of patients was 58.58 ± 10.96 years. The time interval between CT scans was 744.57 ± 205.75 days. Groups A, B, C, and D had intervals of 726.91 days (range: 337–1025 days), 614.14 days (range: 202–1006 days), 757.51 days (range: 420–1125 days), and 867.25 days (range: 590–1280 days), respectively. The duration of radiation therapy was 57.18 days and 56.91 days for groups B and D, respectively (Table 1).

Underlying factors, such as body mass index (BMI) (*p* = 0.9584), hypertension (*p* = 0.9042), diabetes (*p* = 0.7203), and smoking (*p* = 0.6804), might have affected the quality of recipient blood vessels. However, there was no significant difference in the proportion of patients with underlying diseases among groups.

**Figure 4:**
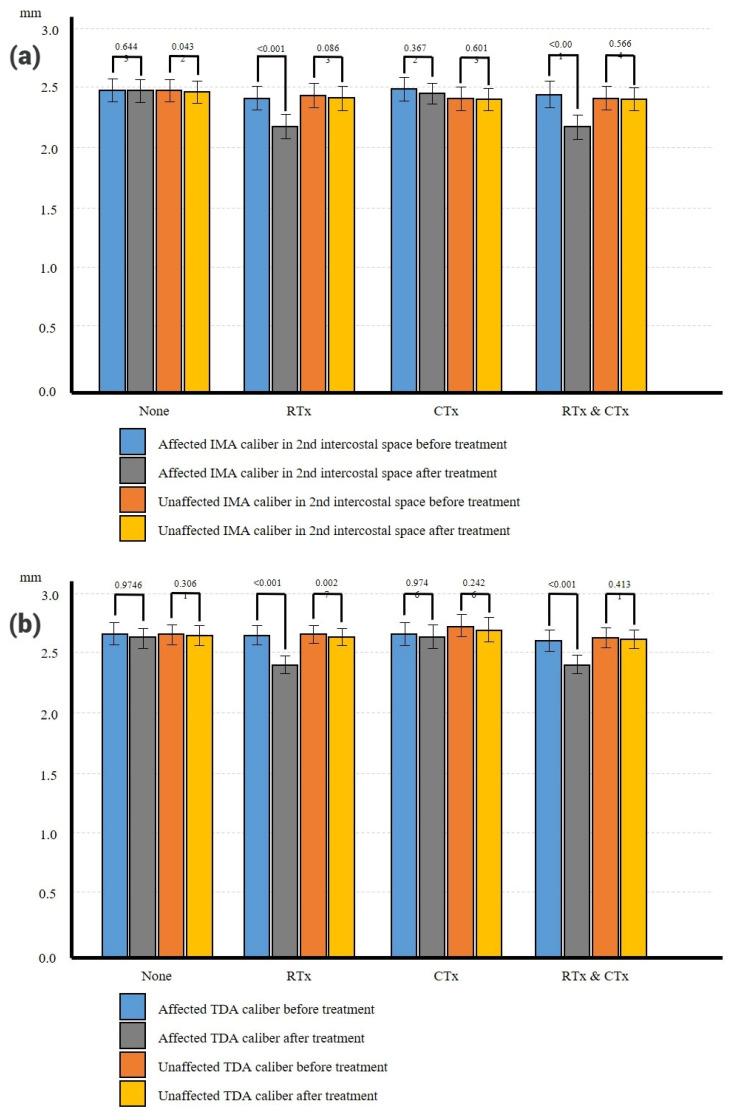
Differences in vessel diameters by group (mm) (IMA and TDA) (**a**) Affected IMA in RTx-treated group and RTx- & CTx-treated group exhibited a significant decrease. IMAs in other groups showed minimal changes following treatment. (**b**) Affected IMA in RTx-treated group and RTx- & CTx-treated group exhibited a significant decrease. IMAs in other groups showed minimal changes following treatment. IMA, internal mammary artery; TDA, thoracodorsal artery.

**Figure 5:**
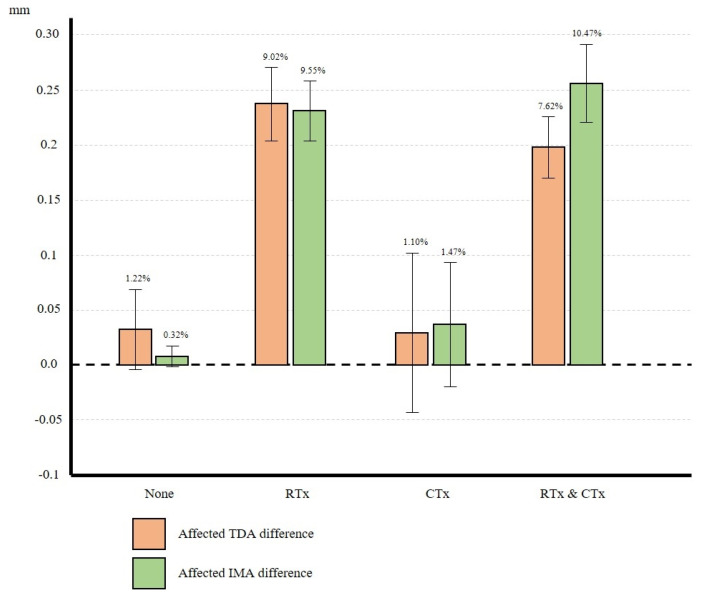
Percentage differences in the diameters of affected vessels in each group (IMA and TDA) IMA, internal mammary artery; TDA, thoracodorsal artery.

In group A, the preoperative mean diameters of the TDA and IMA on the affected side were 2.65 ± 0.27 mm and 2.48 ± 0.28 mm, respectively. On the unaffected side, these diameters were 2.65 ± 0.24 mm and 2.47 ± 0.27 mm, respectively. Postoperatively, the diameters of the TDA and IMA on the affected side were 2.62 ± 0.24 mm and 2.48 ± 0.28 mm, respectively. On the unaffected side, these diameters were 2.63 ± 0.23 mm and 2.47 ± 0.27 mm, respectively (Table 2). No significant change was observed in either diameter on either side (Figure 4 and Figure 5 and Table 3).

In group B, the preoperative diameters of the TDA and IMA on the affected side were 2.63 ± 0.26 mm and 2.42 ± 0.32 mm, respectively. On the unaffected side, these respective diameters were 2.64 ± 0.25 mm and 2.43 ± 0.32 mm. After radiation therapy, the diameters of the TDA and IMA on the affected side were 2.40 ± 0.25 mm and 2.19 ± 0.33 mm, respectively. On the unaffected side, these diameters were 2.63 ± 0.24 mm and 2.42 ± 0.34 mm, respectively (Table 2). There was no significant change in either diameter on the unaffected side. On the other hand, these diameters on the affected side decreased significantly post-radiation therapy by 9.02% and 9.55%, respectively (both *p* < 0.001). Therefore, the diameter of TDA and IMA on the unaffected and affected sides showed no significant differences (Figure 4 and Figure 5 and Table 3). These results indicate significant differences in vessel diameter on the affected side of patients receiving radiation but not chemotherapy, suggesting the potential impact of radiation therapy on blood vessels.

In group C, the preoperative diameters of the TDA and IMA on the affected side were 2.65 ± 0.30 mm and 2.49 ± 0.32 mm, respectively. On the unaffected side, these respective diameters were 2.71 ± 0.30 mm and 2.41 ± 0.33 mm. After chemotherapy, the diameters of the TDA and IMA on the affected side were 2.62 ± 0.33 mm and 2.45 ± 0.29 mm, respectively. Those on the unaffected side were 2.68 ± 0.33 mm and 2.40 ± 0.30 mm, respectively (Table 2). There was no significant difference in either diameter on either side pre- and post-chemotherapy (Figure 4 and Figure 5 and Table 3). These results indicate that chemotherapy had minimal impact on blood vessels.

In group D, the preoperative diameters of the TDA and IMA on the affected side were 2.59 ± 0.30 mm and 2.44 ± 0.38 mm, respectively. On the unaffected side, the diameters of the TDA and IMA were 2.62 ± 0.28 mm and 2.42 ± 0.34 mm, respectively. After treatment, these diameters were 2.40 ± 0.27 mm and 2.19 ± 0.35 mm, respectively. On the unaffected side, the diameters of the TDA and IMA was 2.60 ± 0.27 mm and 2.41 ± 0.33 mm, respectively (Table 2). Comparing the vessel diameters before and after combined radiation and chemotherapy, the diameters of both the TDA and IMA decreased significantly (*p* < 0.001). Notably, the diameters of the TDA and IMA on the affected side decreased by 7.62% and 10.47%, respectively, indicating a larger decrease in diameter of the IMA than that of the TDA (*p* = 0.001) (Figure 4 and Figure 5 and Table 3). There was no significant change in vessel diameter on the unaffected side after the adjuvant treatment.

## 4. Discussion

Adjuvant chemo- or radiotherapy is pivotal in breast cancer management because it can reduce mortality and recurrence rates by 10–25% [12,13]. Therefore, patients who wish to undergo breast reconstruction often require adjuvant treatment. The quality of the recipient and donor vessels is crucial in microsurgical breast reconstruction; therefore, the impact of adjuvant therapy on these vessels is critical for plastic surgeons, especially during delayed breast reconstruction. However, only a few studies have been conducted in this research area.

The risk of free flap failure or revision surgery increases with radiation. A previous study involving 18,776 flaps in 17,532 patients found that preoperative radiation therapy was significantly associated with higher rates of total and partial flap failures (odds ratio = 1.675, 2.161; *p* < 0.001) [14]. Similarly, in a study of 1025 breast reconstruction flaps, the irradiated group exhibited a higher prevalence of vascular complications compared with the non-irradiated group (9.6% vs. 17.3%; *p* = 0.001) [15].

Nevertheless, the precise pathophysiological mechanisms underlying the relationship between radiation therapy and flap failure remain unclear. A previous study has indicated that the increased incidence of free flap failure post-radiation therapy might be due to endothelial dysfunction of vessels caused by radiation. In a study using electron microscopy, irradiated vessels showed relatively thickened vessel walls, intimal dehiscence, fibrin deposition, microthrombi, and endothelium cell dehiscence, which increase the failure rate of free flap surgery due to multiple thrombi [16]. Coronary heart disease and atherosclerosis are also likely to develop following endovascular radiation, primarily due to radiation-induced errors in the mechanisms of programmed cell death [17].

Notably, only a few studies have analyzed the relationship between radiation and vessel diameter. One study has documented a significant decrease in the diameter of the internal carotid artery among patients who underwent irradiation, increasing the risk of stroke-induced brain injury [18]. This suggests that radiation therapy can induce atherosclerotic-like changes in the internal carotid artery, potentially reducing vascular diameter.

In another study involving 40 patients with head and neck cancer treated with an average radiation dose of 6520 cGy over 10 years, post-radiation complications included a notable increase in carotid arterial stenosis, with 16 (40%) of these patients exhibiting significant stenosis. Patients with and without significant stenosis were similar in age, radiation dose, tobacco use, comorbidities, and post-radiation interval (*p* = not significant). Six patients (15%) experienced unilateral complete carotid occlusion, and six (15%) had significant bilateral carotid stenosis. Additionally, three patients (7.5%) suffered a stroke following radiation therapy [19]. Notably, only one study has examined the changes in the diameter of IMA following radiation therapy [19]. A CT angiography study conducted an average of 22 months post-radiation therapy showed a significant decrease in the diameter of the irradiated IMA compared with that of the non-irradiated IMA. Specifically, after radiation exposure, the mean diameter of the irradiated IMAs was 2.39 ± 0.50 mm in the first intercostal space and 1.77 ± 0.46 mm in the second intercostal space. On the non-irradiated side, these values were 2.67 ± 0.49 mm (*p* < 0.001) and 2.11 ± 0.42 mm (*p* < 0.001), respectively. Notably, the study did not directly measure changes in blood vessel diameter pre- and post-radiation therapy in the same patients. Instead, they compared the diameters of blood vessels on the affected side with those of the vessels on the contralateral side in patients who had previously received unilateral radiation therapy. In contrast, our study addressed this limitation by measuring the diameters of corresponding vessels pre- and post-radiation, directly evaluating the changes in vessels after radiation therapy, exerting clinical relevance.

In addition, in a histologic examination study, rabbits injected with high-dose cisplatin exhibited minimal intimal denudation, disarray of smooth muscle cells, and edema of the tunica media [20]. In a study involving 16 patients who underwent preoperative intraarterial chemotherapy with cisplatin and free flap reconstruction, no significant increase in free flap complications was observed after chemotherapy [21]. Furthermore, other studies indicated that chemotherapy increases the risk of atherosclerosis and cardiovascular complications, including ischemic injury and thrombosis. Chemotherapeutic agents such as angiogenesis inhibitors, alkylating agents, antimetabolites, anti-microtubules, and proteasome inhibitors affect nitric oxide and reactive oxygen levels and cause vasospasm, leading to a decrease in vessel diameter [22].

In our study, we observed no significant difference in vessel diameter among patients who underwent only chemotherapy. However, chemotherapy and radiation therapy resulted in a significant decrease in the diameters of both blood vessels. Notably, the degree of decrease in diameter was similar in IMA and TDA in patients who underwent only radiation therapy; In patients who underwent concurrent chemotherapy and radiation therapy, the degree of decrease in diameter was greater for IMA compared with TDA (10.47% vs. 7.62%). This suggests that concurrent radiation and chemotherapy exerts more serious adverse effects on the quality of the IMA than the TDA.

From a microsurgical perspective, the IMA is often chosen as the recipient for DIEP and similar free flaps because its diameter is usually similar to that of the deep inferior epigastric systems, which makes end-to-end anastomosis relatively simple. In patients who received radiotherapy, a reduction of about 7–10% in IMA diameter can create a greater size difference between the recipient and donor vessels, especially if the deep inferior epigastric systems remain unchanged. This size discrepancy may make the anastomosis more technically demanding, disturb blood flow, and increase the thrombus formation and reduced vessel patency in the free flap reconstruction. For these reasons, careful preoperative imaging and meticulous intraoperative assessment of irradiated IMAs are important when planning DIEP-based microsurgical breast reconstruction.

Several recipient vessel options are available for microsurgical breast reconstruction, including the internal mammary, thoracodorsal, circumflex scapular, and internal mammary perforator systems [23]. The circumflex scapular artery and veins have been reported as reliable recipients with favorable caliber and low complication rates, and they are often less affected by tangential chest wall irradiation [24]. However, the circumflex scapular pedicle lies in a relatively deep anatomical plane, which can make dissection more demanding in fibrotic or previously irradiated axillae. By contrast, the thoracodorsal artery is usually more superficial and readily accessible, and has therefore remained our preferred axillary recipient in routine practice. In addition, the thoracodorsal system can support reverse-flow perfusion strategies that provide additional options in complex or previously treated axillary fields, further underscoring its versatility as a recipient vessel in breast microsurgical reconstruction [25]. Taken together, both the thoracodorsal and circumflex scapular vessels are viable recipient options, and the optimal choice should be individualized according to prior radiation fields, local anatomy, and the reconstructive plan.

This study has several limitations. First, it is a retrospective, single-center cohort study that may be subject to selection bias and unmeasured confounding. Second, different combinations of chemotherapy might have different effects on blood vessels, and the interval between pre- and post-treatment CT scans differed among groups, with a notably longer mean interval in group D, reflecting the clinical practice of performing follow-up CT after completion of both chemotherapy and radiotherapy; therefore, our results may underestimate regimen-specific vascular effects, and further research is needed to explore the potential vascular chemical reactions induced by individual chemotherapeutic agents and combinations, as well as to formally adjust vessel diameter changes for CT interval using more robust multivariable analyses in larger, prospective cohorts.

Third, we did not have sufficiently detailed clinical data to directly correlate vessel diameter changes with microsurgical outcomes. This is because most free flap-based autologous reconstructions at our institution were performed as immediate procedures, making it difficult to isolate and quantify the additional impact of adjuvant treatments on clinical outcomes such as thrombosis or flap failure. Therefore, further studies focusing on delayed reconstruction in patients who have received adjuvant treatment are needed to clarify how these treatment-related vessel changes translate into clinical outcomes. Finally, the recipient vessels in the affected area (especially the thoracodorsal artery) are susceptible to various influences such as surgeon technique (e.g., use of energy devices) and intraoperative conditions, which may act as potential confounding factors in our analysis.

## 5. Conclusions

Radiation therapy significantly reduces the diameters of recipient vessels. Therefore, when performing free flap surgery for delayed breast reconstruction, it is crucial to consider the implications of previous adjuvant therapy on affected vessels. Consequently, surgeons can help patients with shared decisions by providing detailed information about the operative risk associated with treatments.

## Figures and Tables

**Table 1:** Patient demographics in each group.

	Group A, *n* (%)	Group B, *n* (%)	Group C, *n* (%)	Group D, *n* (%)	*p*-Value
No. patients	33	44	43	47	
Age (year)	58.4	59.1	59.1	57.7	0.9669
CT interval (day)	726.9	614.1	757.5	867.3	0.0001
RTx duration (day)	-	26.0	-	32.4	0.0001
BMI (Kg/m^2^)	24.9	24.9	24.7	24.5	0.9584
Diabetes mellitus	2(6.1)	6(13.6)	6(14.0)	5(10.6)	0.7203
Hypertension	12(36.4)	16(36.4)	14(32.6)	19(40.4)	0.9042
Smoking	2(6.1)	2(4.5)	1(2.3)	4(8.5)	0.6804

CT, computed tomography; RTx, radiation therapy.

**Table 2:** Measurement of vessel diameters in each group.

			Group A	Group B	Group C	Group D
Affected side	Before treatment	TDA	2.653	2.634	2.653	2.594
(mm)		IMA	2.484	2.416	2.487	2.442
	After treatment	TDA	2.620	2.396	2.624	2.396
		IMA	2.476	2.186	2.451	2.186
Unaffected side	Before treatment	TDA	2.646	2.644	2.711	2.616
(mm)		IMA	2.481	2.434	2.412	2.415
	After treatment	TDA	2.632	2.626	2.683	2.605
		IMA	2.470	2.420	2.405	2.410

IMA, internal mammary artery; TDA, thoracodorsal artery.

**Table 3:** Statistical differences in vessel diameters by group.

		Group A	Group B	Group C	Group D
Affected side	TDA difference (*p*-value)	0.032 (0.975)	0.238 (<0.001)	0.029 (0.975)	0.198 (<0.001)
	IMA difference (*p*-value)	0.008 (0.644)	0.231 (<0.001)	0.037 (0.367)	0.256 (<0.001)
Unaffected side	TDA difference (*p*-value)	0.014 (0.306)	0.018 (0.003)	0.028 (0.243)	0.011 (0.413)
	IMA difference (*p*-value)	0.011 (0.043)	0.015 (0.086)	0.007 (0.601)	0.063 (0.566)

IMA, internal mammary artery; TDA, thoracodorsal artery.

## Data Availability

The data supporting the findings of this study are not publicly available due to privacy and institutional policy restrictions. Requests for access to the data should be directed to the first author, Jong Yun Choi, MD., Ph.D. (jongparry@naver.com).

## Abbreviations

CTx	Chemotherapy
RTx	Radiotherapy
CT	Computed Tomography
TDA	Thoracodorsal artery
IMA	Internal mammary artery
